# Pregnancy and liver adenoma management: PALM-study

**DOI:** 10.1186/1471-230X-12-82

**Published:** 2012-06-29

**Authors:** Susanna M van Aalten, Mirelle E E Bröker, J J V Busschbach, Harry J de Koning, Robert A de Man, Eric A P Steegers, Ewout W Steyerberg, Turkan Terkivatan, Jan N M IJzermans

**Affiliations:** 1 Departments of Surgery, Erasmus Medical Center, P.O. Box 2040, 3000 CA, Rotterdam, The Netherlands; 2 Psychology and Psychotherapy, Erasmus Medical Center, P.O. Box 2040, 3000 CA, Rotterdam, The Netherlands; 3 Public Health, Erasmus Medical Center, P.O. Box 2040, 3000 CA, Rotterdam, The Netherlands; 4 Hepatogastroenterology, Erasmus Medical Center, P.O. Box 2040, 3000 CA, Rotterdam, The Netherlands; 5 Obstetrics and Gynaecology, Erasmus Medical Center, P.O. Box 2040, 3000 CA, Rotterdam, The Netherlands

## Abstract

**Background:**

Hepatocellular adenoma (HCA) in pregnant women requires special considerations because of the risk of hormone induced growth and spontaneous rupture, which may threaten the life of both mother and child. Due to scarcity of cases there is no evidence-based algorithm for the evaluation and management of HCA during pregnancy. Most experts advocate that women with HCA should not get pregnant or advise surgical resection before pregnancy. Whether it is justified to deny a young woman a pregnancy, as the biological behavior may be less threatening than presumed depends on the incidence of HCA growth and the subsequent clinical events during pregnancy.

We aim to investigate the management and outcome of HCA during pregnancy and labor based on a prospectively acquired online database in the Netherlands.

**Methods/design:**

The Pregnancy And Liver adenoma Management (PALM) - study is a multicentre prospective study in three cohorts of pregnant patients. In total 50 pregnant patients, ≥ 18 years of age with a radiologically and/or histologically proven diagnosis of HCA will be included in the study. Radiological diagnosis of HCA will be based on contrast enhanced MRI. Lesions at inclusion must not exceed 5 cm. The study group will be compared to a healthy control group of 63 pregnant patients and a group of 63 pregnant patients with diabetes mellitus without HCA. During their pregnancy HCA patients will be closely monitored by means of repetitive ultrasound (US) at 14, 20, 26, 32 and 38 weeks of gestation and 6 and 12 weeks postpartum. Both control groups will undergo US of the liver at 14 weeks of gestation to exclude HCA lesions in the liver. All groups will be asked to fill out quality of life related questionnaires.

**Discussion:**

The study will obtain information about the behaviour of HCA during pregnancy, the clinical consequences for mother and child and the impact of having a HCA during pregnancy on the health related quality of life of these young women. As a result of this study we will propose a decision-making model for the management of HCA during pregnancy.

**Trial registration:**

Dutch trial register: NTR3034

## Background

Hepatocellular adenoma (HCA) is rare benign tumor of the liver that occurs particularly in women during their reproductive years. The incidence is not exactly known. Studies performed years ago show an estimate incidence of 1-1.3 per 1,000,000 in women who have never used oral contraceptives (OC), compared to 30-40 per 1,000,000 in long-term users [1,2]. The association of HCA with the use of OC was first described in 1973 [3]. In subsequent years many authors have supported the hypothesis of an association between OC and HCA [4-8]. The mechanism by which estrogen or other steroids contributes to the development of HCA is still not understood and studies are rare. Symptomatic patients with HCA present with right upper quadrant abdominal pain or discomfort secondary to bleeding within the HCA, elevated liver enzymes and symptoms of life treating hemorrhage into the peritoneal cavity. However, most patients with HCA are asymptomatic and present as an incidental finding during ultrasonographic examination of the abdomen for unrelated reasons or are noted during laparoscopic cholecystectomy. Despite its benign nature, the diagnosis of HCA has a great impact of the lives of these, mostly, young women because HCA can be complicated by hormone induces growth and rupture. Besides that malignant transformation of HCA into hepatocellular carcinoma has been reported with an overall frequency of 4.2% [9].

Regardless of the exact etiology and risk factors all female patients should be advised to stop OC’s and other hormone medication such as hormone replacement therapy, since regression of HCA may occur when steroids are withdrawn [10-13] and observation should be the first choice of treatment for most patients with HCA. Because of the risk for spontaneous rupture most authors believe that surgical resection is required if the diameter exceeds 5 cm after 6 months of follow-up without OC use, if the lesion does not show adequate regression after discontinuation of OC or if rebleeding occurs [14-17]. Surgical resection is also indicated if there is diagnostic doubt e.g. whether a tumor is malignant [18,19].

HCA in pregnant women requires special considerations because of the risk of hormone induced growth and spontaneous rupture, due to increased levels of steroid hormones during pregnancy that may threaten the life of both mother and child. Most experts advocate that women with HCA should not get pregnant or advise surgical resection before pregnancy [2,18]. Cobey et al. reported a maternal and fetal mortality risk of ruptured HCA during pregnancy of 44% and 38%, respectively [20]. However, all these cases were published in the 1970s and 1980s and nowadays the introduction and widespread use of highly advantage imaging modalities have probably decreased the doctors’ delay in the diagnosis of HCA. We recently proposed not to discourage all women with HCA from pregnancy, based on a study in which we monitored twelve women with documented HCA during a total of 17 pregnancies. In 4 cases HCA’s grew during pregnancy, requiring a Caesarean section in 1 patient (2 pregnancies) and radiofrequency ablation (RFA) in 1 case during the first trimester of pregnancy. All pregnancies had an uneventful course with a successful maternal and fetal outcome [21]. However, there is no evidence-based algorithm for the evaluation and management of HCA during pregnancy and labor, due to scarcity of cases. The conclusion not to discourage all women with HCA from pregnancy has, however, to be proven in a large multicentre study in which we will closely monitor pregnant patient with a HCA in a prospectively acquired database to give more insight in the behaviour of HCA during pregnancy.

## Methods/design

### Study objective

In this study we will investigate the management and outcome of HCA during pregnancy and labor based on a prospectively acquired online database in the Netherlands.

Main objective of the PALM-study

 • To investigate the incidence of HCA growth during pregnancy and labor.

Secondary objectives of the PALM-study

 • To investigate in which trimester of pregnancy growth of HCA occurs;

 • To investigate the degree of growth of HCA during pregnancy;

 • To investigate whether there is regression of HCA postpartum;

 • To investigate the HCA-related interventions during pregnancy and labor;

 • To investigate the incidence of bleeding of HCA during pregnancy and labor;

 • To investigate liver-related clinical signs during pregnancy;

 • To investigate elevated liver enzymes during pregnancy;

 • To evaluate the health related quality of life of pregnant patients with HCA;

 • To investigate whether there is a difference between health related quality of life of pregnant patients with HCA and pregnant patients with other comorbidity that have an indication for pregnancy care at the obstetrician in secondary care and healthy pregnant patients.

### Study design

The PALM-study is a multi-centre prospective study in three cohorts of pregnant women. The study started on November 1 2011 and inclusion of patients will be a period of minimal 3 to maximal 5 years. In total 50 pregnant patients with HCA < 5 cm will be included in the study. These patients will be compared to a healthy control group consisting of 63 pregnant patients without HCA and a group consisting of 63 pregnant patients with diabetes mellitus (DM). Approval of the Medical Ethics Committee of Erasmus Medical Centre was obtained, NL36058.078.11.

### Patient selection

#### Study group

Properly Dutch speaking, pregnant patients, 18 years of age or older with a radiologically and/or histologically proven diagnosis of HCA can be included in the study. Radiological diagnosis of HCA will be based on contrast enhanced magnetic resonance imaging (MRI) and if available in combination with (contrast enhanced) ultrasonography (US). Lesions must not exceed 5 cm. In the first weeks of pregnancy patients will be referred to the obstetrician for pregnancy care. Baseline starts at 14 (+/−3) weeks of gestation. At this day and every 6 weeks patients will undergo US of the HCA lesion at the radiologist. Before US of the HCA lesions patients will be asked to fill out generic health related quality of life questionnaires (12-item Short Form SF 12 and EuroQol questionnaire EQ-5d), a generic anxiety questionnaire (State-Trait Anxiety Inventory STAI-6) and the Impact of Event Scale (IES) questionnaire for thoughts and feelings about HCA around the US. One week afterwards the study group will be asked to fill out the STAI-6 en IES again. At 14 and 32 weeks of pregnancy patients will undergo venapunction.

#### Control group 1 (healthy pregnant patients without HCA)

Properly Dutch speaking, healthy pregnant patients, 18 years of age or older without HCA.

In the Netherlands, pregnant women will start pregnancy care with an independently practicing midwife early in pregnancy at the primary care level [22]. The midwife is responsible for the pregnant women as long as the pregnancy, labor or postpartum period is normal [23]. In case of complication, the midwife will refer the women to the obstetrician in secondary care [22,23]. Women with a high risk profile based on their medical or obstetric history will be cared for the obstetrician from the start of pregnancy [22,23].

Patients presenting at the practicing midwife will be asked to participate in the study. Thereafter, the patients will be included in the study by the study investigator. Patients will undergo US of the liver at 14 (+/-3) weeks of gestation to exclude HCA lesions in the liver. At this day and every other 6 weeks patients will be asked to fill out the SF-12 and EQ-5d questionnaire. At 14 and 32 weeks of pregnancy patients will undergo venapunction. In case of an uncomplicated pregnancy, the patient remains under the care of her practicing midwife during her pregnancy and postpartum.

#### Control group 2 (pregnant patients with Diabetes Mellitus)

Properly Dutch speaking, pregnant patients, 18 years of age or older with Diabetes Mellitus, can be included in the study. These patients have an indication for pregnancy care at the obstetrician in secondary care. Patients will undergo US of the liver at 14 (+/-3) weeks of gestation to exclude HCA lesions in the liver. At this day and every other 6 weeks patients will be asked to fill out the SF-12 and EQ-5d questionnaire. At 14 and 32 weeks of pregnancy patients will undergo venapunction.

For all groups informed consent is mandatory. A patient can always withdraw her consent at anytime during the study where after she is referred for the present standard of care.

### Hypothesis

Pregnancy may be allowed in case of one or more known HCA < 5 cm (without previous intervention), because a HCA < 5 cm will not disturb the course of pregnancy.

Disrupted course of pregnancy:

 • interventions during pregnancy (radiological and/or surgical intervention).

 • Decreased quality of life and/or anxiety in patients during pregnancy related to the presence of HCA in the liver and possible growth during pregnancy.

### Retrospective cohort study

We have previously reported that more than half of the HCA are discovered after the patient has sustained at least one pregnancy and none of these patients have reported problems during their pregnancies [19]. As mentioned above, recently we described a small but unique series of 12 women with documented HCA who were closely monitored during a total of 17 pregnancies between 2000 and 2009. In 4 cases HCA’s grew during pregnancy, requiring a Caesarean section in 1 patient (2 pregnancies) and RFA in 1 case during the first trimester of pregnancy to treat a hormone sensitive HCA, thereby excluding potential growth later on in pregnancy. No intervention was performed in the other 14 cases. All pregnancies had an uneventful course with a successful maternal and fetal outcome and we concluded that a “wait and see” management may be advocated in pregnant women presenting with HCA. In women with large tumours or in whom HCA had complicated previous pregnancies, surgical resection may be recommend [21]. However, additional data from different centres for the risk of hormone induces growth and rupture of HCA during pregnancy is needed.

### Interventions

During their pregnancy HCA patients will be closely monitored by means of repetitive US (and MRI in case of growth of the lesion) at 14 (+/−3) and 20 and 26 and 32 and 38 weeks of gestation and 6 and 12 weeks postpartum. At the same days both control groups will be asked to fill out the SF-12 and EQ-5d questionnaire at 14 (+/−3) and 20 and 26 and 32 and 38 weeks of gestation and at 6 and 12 weeks postpartum (Fig. 1). The study group will be asked to fill out the SF-12, EQ-5d, STAI-6 and IES questionnaires before and one week after US of the HCA lesion(s). Both control groups will undergo US of the liver at 14 (+/−3) weeks of gestation to exclude HCA lesions in the liver. At 14 and 32 weeks of pregnancy all patient groups will undergo venapunction.

### Online database

We established a website which allows hepatologists, surgeons and gyneacologists to submit clinical data in an online database. Each centre will have a code to log in and patients will be consecutively assessed a unique number. Registration of a new patient includes entry of the following data: date of birth, weight, height, date of hospital admission, symptoms at presentation, known risk factors for HCA such as glycogenosis and familial polyposis, [24] previous pregnancies, previous use of OC or other hormone medication including hormone replacement therapy, course of HCA after discontinuation of OC, size of HCA before pregnancy, size of HCA during pregnancy (14 (+/−3) and 20 and 26 and 32 and 38 weeks), course of HCA postpartum (6 and 12 weeks postpartum), complications and management during pregnancy, gestation time, way of delivery (vaginally, Caesarean section), maternal and fetal outcome, complications and management after delivery. Only authorized users can gain action to the online database of his or her patients. The database offers excess to the registered data on anytime and anywhere. The coordinating investigator will monitor whether all required fields are completed.

### Follow-up

Follow-up of patients takes place at 6 and 12 weeks postpartum postpartum by means of US (and MRI in case of growth) to document the size of HCA postpartum (Table 1). Both control groups will be asked to fill out the SF-12 and EQ-5d questionnaires at these days. The study group will be asked to fill out the SF-12, EQ-5d, STAI-6 and IES questionnaires before and one week after US of the HCA lesion(s).

**Table 1:** Follow-up PALM-study


**Pregnancy**			
**Weeks**	**Ultrasonography**	**Venapunction**	**Questionnaires**
14	S, C1, C2	S, C1, C2	S, C1, C2 *
20	S		S, C1, C2 *
26	S		S, C1, C2 *
32	S	S, C1, C2	S, C1, C2 *
38	S		S, C1, C2 *
**Post-partum**			
**Weeks**	**Ultrasonography**	**Venapunction**	**Questionnaires**
6	S		S, C1, C2 *
12	S		S, C1, C2 *

S, study group; C1 control group 1 (healthy pregnant patients without HCA); C2, control group 2 (pregnant patients with Diabetes Mellitus).

*The study group will be asked to fill out the SF-12, EQ-5d, STAI-6 and IES questionnaires before and one week after US of the HCA lesion(s). Both control groups will be asked to fill out the SF-12 and EQ-5d questionnaire.

### Outcome measures

Primary outcome: Biological behaviour and clinical consequences of HCA < 5 cm during pregnancy. Growth is measured by repetitive US (and MRI in case of growth) at 14 (+/−3) and 20 and 26 and 32 and 38 weeks of gestation.

Secondary outcome: General health and pain scales as a measure for quality of life and anxiety related questionnaires for thoughts and feelings of adenomas around US. Other secondary outcomes are complications due to growth of the HCA during pregnancy possibly followed by interventions during pregnancy, incidence of hemorrhage and rupture of the HCA, incidence of liver-related clinical signs during pregnancy (itch, icterus), incidence of elevated liver enzymes during pregnancy.

### Power calculation

In our previous study we measured growth of HCA in 4 out of 17 pregnancies (24%) or in 3 out of 12 women (25%). On a yearly basis approximately 50 new patients with HCA are seen at the outpatient clinic of the Erasmus University Medical Centre. The expectation is that 5% (5) of these women get pregnant. The expectation is that a total of 50 pregnant HCA patients from different tertiary referral centres in the Netherlands can be included in the study during a period of 3 to maximum 5 years.

A difference of 0.5 Cohen’s D in health-related quality of life is a relevant difference [25]. We calculated that for this purpose 63 patients in both control groups have to be enrolled. A two-sample *t* test was performed with a two-sided significant level of 0.05 and a power of 0.80.

### Access to personal data

Medical data with which the identity of a patient could be traced will be replaced by a code number. The coordinating investigator is the only one who has the key to the code numbers and knows which code number stands for which patient. The principal investigator has only access to the coding system of his or her patients and will never be able to open the database from other centres. Only members of the investigating team and members of the medical ethical committee of the participating centres will have access to the medical data. All data will be collected in a prospectively acquired database by the principal investigators and managed by the coordinating investigator.

## Discussion

Once the diagnosis of HCA has been established, patients will be advised to discontinue OC. Expert opinions are very variable regarding treatment and follow up in complex situations were multiple factors play a role in determining the management strategy, like pregnancy [18].

As to date there are limited data about the behavior of HCA during pregnancy and labor and therefore we cannot identify precisely those at risk for complications. However, in 2006 we reported a series of 48 patients of which in 44% HCA were discovered after the patient had sustained at least one pregnancy [19]. None of these patients have reported problems during their pregnancies. Likely, only a small subgroup of patients may experience complications and to date pregnancy might be discouraged in too many patients caused by unnecessary intervention before pregnancy. We hypothesize that pregnancy may be allowed in case of one or more known HCA < 5 cm (without previous intervention), because HCA < 5 cm will not disturb the course of pregnancy. Close monitoring during pregnancy by means of repetitive US (and MRI in case of growth) should be carried out to rule out rapid growth of the lesion. The risk of rupture seems the highest during the third trimester of pregnancy [20]. Most likely due to the cumulating level of estrogens and an increase in hyperdynamic circulation combined with an increase in vascularity of the liver with growth of the adenoma [20]. Symptoms and the level of liver enzymes will be registered to find out if there is a relation between symptoms, elevated liver enzymes and growth of the HCA during pregnancy. Patients will be followed-up postpartum to investigate if there is a risk of HCA complications after delivery.

The Society of American Gastrointestinal and Endoscopic Surgeons (SAGES) provided guidelines for diagnosis, treatment, and use of laparoscopy for surgical problems during pregnancy [26]. However, these guidelines are mostly based on case reports and retrospective studies and therefore graded at a low level of evidence. The SAGES suggest that MRI without the use of intraveneous gadolinium, and US is considered safe and can be used at any stage of pregnancy (Level IIIB and Level IIA respectively) [26]. Data regarding safety of CEUS during pregnancy is scare and yet uncertain. However, Hua et al. reported an animal study in which SonoVue may affect the placenta [27]. Therefore, we will not use CEUS for patient follow-up during pregnancy.

One should be aware of the potential risks as an intervention may still be indicated during pregnancy. In approximately one in 635 pregnancies a non-obstetric operation during pregnancy is required, especially appendectomy, cholecystectomy and adnexal precedures [28]. However, it is conceivable that more non-obstetric operations might be required due to the risk of hormone induced growth and spontaneous rupture of HCA during pregnancy. Despite maternal and fetal outcomes following abdominal disease and surgery in pregnancy improved over the past years, the exact risk of HCA-related interventions during pregnancy to both mother and fetus is unknown [29]. We do know that changes in physiology and abdominal anatomy characteristics of pregnancy make abdominal surgery more difficult [29]. The least risk of general anaesthesia is in the 2nd trimester of pregnancy [30].

Based on a systematic review of the literature, Wilson et al. suggested angioembolisation and formal resection in case of haemorrhage of HCA during pregnancy and suggested this strategy to be safe for both the mother and the fetus with good clinical outcomes [31]. The role of RFA during pregnancy is not well studied. In our previous study we described a RFA procedure during the first trimester of pregnancy [21] and Fujita et al. reported a pregnant patient with a HCA that was treated by RFA during her second trimester of pregnancy (18^th^ week of gestation) [32].

The influence on the course of pregnancy, since a woman is aware of having a HCA, is also unknown. Patients can get horrified when confronted with the new diagnosis of a hepatic mass [20] and it is conceivable that women can be anxiety during pregnancy due to the presence of HCA in the liver and the possible growth during pregnancy. Therefore, quality of life will be an important measurement for future management of HCA during pregnancy. It is conceivable that frequent monitoring by means of US may comfort the patients or can be frightening. All patient groups will be asked to fill out the SF-12 and EQ-5d questionnaires every 6 weeks. HCA patients will be asked to fill out the STAI-6 and IES questionnaires before the US of the liver lesions and one week after US to investigate anxiety related to HCA and US during pregnancy.

Our main point of interest is whether it is justified to deny a young woman with a HCA < 5 cm a pregnancy. With this study we hope to obtain information about the behaviour of HCA during pregnancy and the impact of HCA during pregnancy on the life of these young women. Furthermore we hope to propose a decision-making model for the management of HCA during pregnancy.

## Abbreviations

HCA: Hepatocellular Adenoma; US: Ultrasound; OC: Oral Contraceptives; DM: Diabetes Mellitus; MRI: Magnetic Resonance Imaging; SF 12: 12-item Short Form; EQ-5d: EuroQol questionnaire 5d; STAI-6: State-Trait Anxiety Inventory; IES: Impact of Event Scale; SAGES: Society of American Gastrointestinal and Endoscopic Surgens; CEUS: Contrast Enhanced Ultrasound; RFA: Radiofrequency ablation.

## Competing interests

The authors declare that they have no competing interests.

## Authors’ contribution

SMA and MEEB are responsible for the drafting of the manuscript and study design, these authors contributed equally to this work. JJB, HJDK, RADM, EAPS, EWS, TT and JNMIJ are responsible for the study design and revision of the manuscript. All authors have read and approved the manuscript.

## Funding

Stichting Coolsingel

Fonds NutsOhra

## Dutch Liver Surgery Study Group

T.M. van Gulik, MD, PhD *Academic Medical Center, Amsterdam, Department of Surgery*

R. van Hillergersberg, MD, PhD *University Medical Centrer Utrecht, Department of Surgery*

C.H.C. de Jong*,* MD, PhD *Academic Hospital Maastricht, Department of Surgery*

J. Klaase, MD *Medical Spectrum Twente, Department of Surgery*

M.S.L. Liem, MD *Deventer Hospital, Department of Surgery*

R.J. Porte, MD, PhD *University Medical Center Groningen, Department of Surgery*

A.M. Rijken, MD *Amphia Hospital Breda, Department of Surgery*

R.M.H. Roumen, MD *Máxima Medical Center Veldhoven, Department of Surgery*

A.F.M. Schaapherder, MD*Leiden University Medical Center, Department of Surgery*

M.P. van den Tol, MD *VU Medical Center Amsterdam, Department of Surgery*

J.H.W. de Wilt, MD, PhD *University Medical Center St. Radboud, Department of Surgery*

## Pre-publication history

The pre-publication history for this paper can be accessed here:

http://www.biomedcentral.com/1471-230X/12/82/prepub
